# Oral Cancer Therapy: The Importance of Adherence

**Published:** 2017-11-01

**Figure 1:**
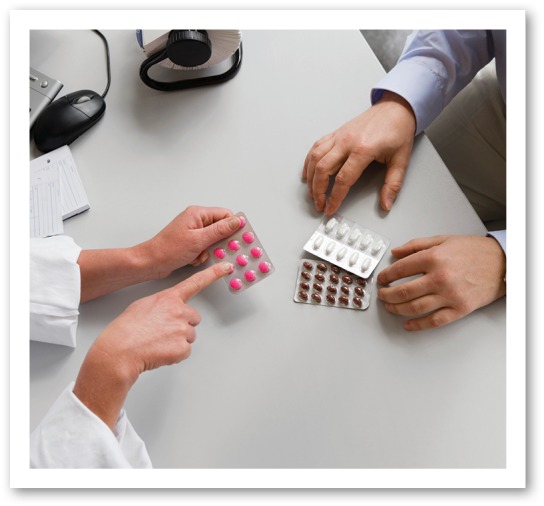


When people think of cancer treatment, they frequently picture patients receiving intravenous (IV) treatments over several hours in a hospital or clinic setting. But over the past 2 decades, more and more cancer treatments that can be taken at home—in tablet, capsule, or liquid form—have become available. 

**A Common Struggle**

Oral cancer treatments are easier to take, and surely more convenient than having to be at a clinic each time you need to receive a treatment. However, despite the many advantages of oral treatments, patients often struggle with adherence—taking oral medications exactly as directed by a healthcare provider. Why is this important? Simply put, not taking prescribed medications for cancer treatment as directed can decrease how well they work against your cancer. 

In these pages, you’ll find some strategies to help you take your cancer drugs properly and safely.

**Understanding Your Treatment**

Before you start your oral cancer treatment, you should talk with your healthcare provider to be sure you know the answers to the following questions:

- Where should I get my prescription filled?
- What should I do if I can’t afford my medicine?
- How should I store my medicine?
- When should I take my medicine? Every day? Twice a day?
- Are there any special instructions for taking my medication? (Can I break the pills? Can I chew them?)
- Does it matter when I take it (morning, bedtime, etc.)?
- Should I take the drug with food, or on an empty stomach? Are there any foods to avoid?
- Will other drugs, supplements, or vitamins I’m taking affect how the cancer drug works?
- What should I do if I miss a dose?
- What side effects might occur? How should I handle them?
- How do I know when to call if I don’t feel well after taking my medicine?
- Who should I call with questions?

**Tips for Remembering to Take Your Medications**

Whether you have an active life or are fairly sedentary, remembering to take your cancer medication can be more of a challenge than one might imagine. Despite the fact that you may have the best intentions, we’re all human, and life sometimes gets in the way. 

But there are many strategies and devices available to help you remember to take your medication, whether you’re at home or traveling. Some are simple, and some are rather high-tech. Choose the method that suits your style best. And remember, if it doesn’t work, try another method to eventually find the strategy that works for you. 

The Benefits of a Routine

It may sound obvious, but a key concept for better medication adherence is creating a routine that helps you remember to take your medications at the same time each day. If you need to take your medicine every morning before breakfast, consider pairing it with another activity you know you won’t forget to do: If you make coffee every morning, you may want to keep your medicine right next to the coffee maker so it becomes routine to do the two activities together. Some people keep their medications next to their toothpaste for the same reason.

**Table 1:**
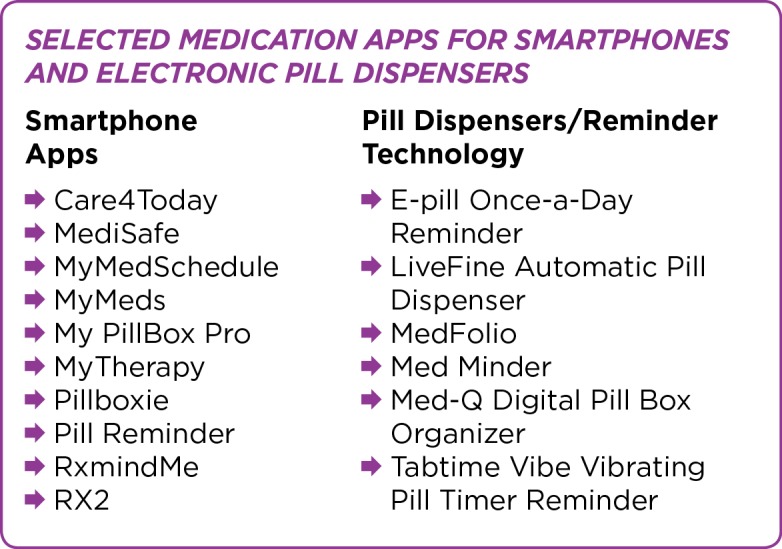
SELECTED MEDICATION APPS FOR SMARTPHONES AND ELECTRONIC PILL DISPENSERS

Reminders Are Key!

Sticking to a schedule also contributes to adherence. Are you a visual person? Keep a wall calendar with the days you need to take your medication clearly marked. Check off the task on the calendar as soon as you take it, as once the day gets going it can be hard to remember if you took your medication or not, especially if you have other medications to consider.

Some people benefit from a pillbox organized by the days of the week. Just remember to keep the pillbox visible (assuming it’s safe from the reach of children or pets). You can get a simple one at your local drugstore, or you can try an electronic pill box that you will alert you to take your medications at certain times. If you’re comfortable using a smartphone, electronic reminders—including cell phone alarms, smartphone apps, and text messages—could work well for you. You’ll find a few examples of electronic reminder technology in the table above.

**Figure 2:**
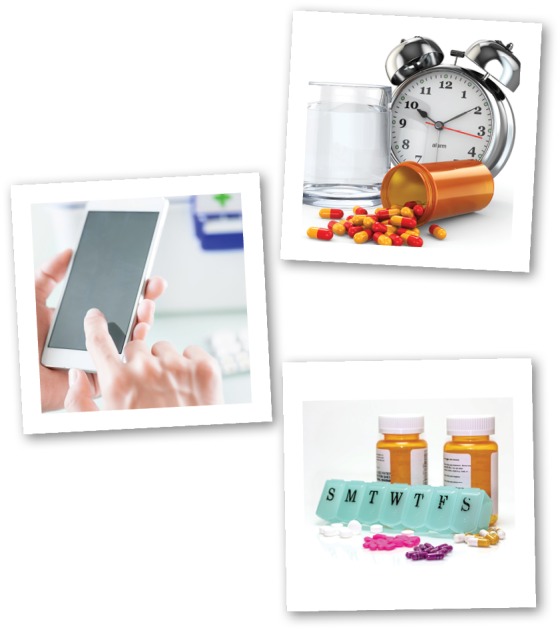


The Buddy System

Consider asking a family member or friend to give you regular reminders to take your medicine. This can be handy when your normal routine is disrupted by traveling. A quick phone call each morning can be a helpful source of support until you can get back to familiar surroundings. 

**Bottom Line: Communication**

Regardless of the method you choose to help you adhere to your cancer medication schedule, communication is key. If you find that it’s a challenge for you to take your medications, for whatever reason, talk it over with your healthcare provider. It’s crucial for them to know whether you’re taking your medications correctly. As a team, you can work on finding the right strategy for you to succeed in taking your medications properly, and ultimately getting the most out of your treatment. 

